# Effect of community-based distribution of misoprostol on facility delivery: a scoping review

**DOI:** 10.1186/s12884-019-2539-5

**Published:** 2019-11-06

**Authors:** Gizachew Tadele Tiruneh, Bereket Yakob, Wubegzier Mekonnen Ayele, Muluneh Yigzaw, Meselech Assegid Roro, Araya Abrha Medhanyi, Etenesh Gebreyohannes Hailu, Yibeltal Tebekaw Bayou

**Affiliations:** 1 JSI Research & Training Institute, Inc./ The Last Ten Kilometers (L10K) Project, Addis Ababa, Ethiopia; 2 Members of the National Reproductive, Maternal, Newborn, Child, Adolescent Health, and Nutrition (RMNCAH-N) Research Advisory Council (RAC), Addis Ababa, Ethiopia; 3 Department of Global Health and Population /Fenot Project, Harvard T.H. Chan School of Public Health, Addis Ababa, Ethiopia; 4 Addis Ababa University School of Public Health, Addis Ababa, Ethiopia; 5 Jhpiego/HRH Project, Addis Ababa, Ethiopia; 6 School of Public Health, College of Health Sciences, Mekelle University, Mekelle, Ethiopia; 7 Federal Ministry of Health, Addis Ababa, Ethiopia

**Keywords:** Community-based distribution, Diversion of facility birth, Facility delivery, Misoprostol

## Abstract

**Introduction:**

Community distribution of misoprostol to pregnant women in advance of labor is one of the compelling strategies for preventing postpartum hemorrhage. Concerns have been reported that misoprostol distribution could reduce facility delivery or lead to misuse of the medication. This scoping review was conducted to synthesize the evidence on the effect of community-based misoprostol distribution on rates of facility delivery, and to assess the frequency of mothers taking distributed misoprostol before delivery, and any harmful outcomes of such misuse.

**Methods:**

We included peer-reviewed articles on misoprostol implementation from PubMed, Cochrane Review Library, Popline, and Google Scholars. Narrative synthesis was used to analyze and interpret the findings, in which quantitative and qualitative syntheses are integrated.

**Results:**

Three qualitative studies, seven observational studies, and four experimental or quasi-experimental studies were included in this study. All before-after household surveys reported increased delivery coverage after the intervention: ranging from 4 to 46 percentage points at the end of the intervention when compared to the baseline. The pooled analysis of experimental and quasi-experimental studies involving 7564 women from four studies revealed that there was no significant difference in rates of facility delivery among the misoprostol and control groups [OR 1.011; 95% CI: 0.906–1.129]. A qualitative study among health professionals also indicated that community distribution of misoprostol for the prevention of postpartum hemorrhage is acceptable to community members and stakeholders and it is a feasible interim solution until access to facility birth increases.

In the community-based distribution of misoprostol programs, self-administration of misoprostol by pregnant women before delivery was reported in less than 2% of women, among seven studies involving 11,108 mothers. Evidence also shows that most women who used misoprostol pills, used them as instructed. No adverse outcomes from misuse in either of the studies reviewed.

**Conclusions:**

The claim that community-based distribution of misoprostol would divert women who would have otherwise had institutional deliveries to have home deliveries and promote misuse of the medication are not supported with evidence. Therefore, community-based distribution of misoprostol can be an appropriate strategy for reducing maternal deaths which occur due to postpartum hemorrhages, especially in resource-limited settings.

## Introduction

Maternal mortality ratios (MMR) remain high in low-and-middle-income countries (LMICs), and reduction of MMR continues to be a priority challenge in the Sustainable Development Goals (SDG) era [1]. Accordingly, to achieve the SDG of reducing the global MMR to 70 per 100,000 live births by 2030, LMICs needs to implement innovative and high impact interventions aimed at preventing and managing the main causes of maternal deaths and providing high-quality services in the continuum of maternity care [2, 3].

A wealth of evidence shows that hemorrhage is one of the major causes of maternal mortality [4–7]. More than two-thirds of maternal deaths due to hemorrhage occur during the postpartum period, which accounts for 20% of all maternal deaths in developing regions [5]. However, in Ethiopia, a systematic review of national evidence shows that PPH accounts for 30% of maternal deaths [8, 9].

Most maternal deaths occurring due to PPH are in poorly resourced facilities or outside of a health facility where there is no access to skilled obstetric care [10–12]. Women who deliver at home face the highest risk of PPH, as they do not benefit from the support of skilled birth attendants and are less likely to receive timely care and medications that prevent and manage PPH [12]. Evidence shows that most PPH-associated deaths could be avoided if active management of third stage of labor (AMTSL) is implemented [13], adverse outcomes and complications are prevented or managed using safe drugs in communities and facilities, and effective referral mechanisms are implemented during delivery and in the postpartum period [14]. Intravenous or intramuscular administration of uterotonics are the most essential component of AMTSL [15] and oxytocin remains the first choice uterotonics for the prevention of PPH [14].

Misoprostol distribution at community level to women during pregnancy is one of the interventions for preventing PPH to reach women who deliver at home without skilled attendant [16–18]. Misoprostol is a generic, inexpensive, heat-stable, and potent uterotonic that can be administered orally, sublingually, rectally, and vaginally [19] for the prevention of PPH. It has considerable advantages over other uterotonics in resource-poor settings to reach woman without access to institutional delivery. Misoprostol has been studied in different setups and is endorsed by the World Health Organization (WHO) as a solution for women who give birth in facilities without oxytocin or where there is low coverage of skilled attendance [16]. Clinical trials have verified the effectiveness and safety of community distribution of misoprostol [14, 17, 20, 21] where access to skilled birth attendance and oxytocin is limited. A pooled estimate of randomized controlled trials (RCT) comparing 600 μg of oral or sublingual misoprostol with placebo in primary care or home delivery settings show that misoprostol resulted in 24 and 41% reductions in the incidence of PPH and severe PPH compared with placebo, respectively [17].

Despite the existing evidence, community-based distribution of misoprostol is still the least prioritized intervention in the maternal survival strategies [18, 22–25]. This is due to concerns of policymakers’ and practitioners’ [12, 17, 21, 25] that misoprostol distribution at community level might decrease facility deliveries, possibly lead to misuse of misoprostol (including taking the drug before delivery, and using the drug for the purpose of inducing abortion), and lack of technologies and expertise to diagnose multiple pregnancies before using it at community levels in resource-limited settings [20, 26]. A range of other barriers at the health system, community, and policy levels are also impeding access to misoprostol for prevention of PPH. These barriers include: 1) absence of registration of misoprostol for the management of PPH [25, 27], 2) fear and apprehensions of providers and policymakers regarding its use [25, 27], 3) lack of evidence-based guidelines and provider training [25], 4) inadequate staffing and lack of knowledge and skill of providers regarding causes of PPH, and 5) limited knowledge of the community regarding the appropriate dosage and timing of administration for PPH presentation and management [20, 27].

This scoping review was, therefore, conducted to synthesize the evidence on the effect of community-based misoprostol distribution in advance of delivery on rates of facility delivery, and to assess the frequency of mothers taking distributed misoprostol before delivery, and any harmful outcomes of such misuse.

## Methods

### Criteria for inclusion

In this study, researchers used a scoping review methodology to get a wide range of information from both qualitative and quantitative studies. All types of literature on community-distribution of misoprostol for the prevention of PPH reported in English language were included, with no specification on timing of publication.

### Search strategy

We identified peer-reviewed articles on implementation of community distribution of misoprostol from PubMed, Cochrane Review Library, Popline, and Google Scholars which were made available from February 1–15, 2019. We also applied a snowball approach of searching from the references of papers of the initial search.

The following search strategy was used to search literature from PubMed and CENTRAL databases;

“((((((((((((Africa OR Asia OR Caribbean OR West Indies OR South America OR Latin America OR Central America OR Middle East))) OR ((developing countr* OR less developed country * OR under developed country * OR underdeveloped country * OR middle income country * OR low income countr*)))))) AND ((((postpartum hemorrhage) OR post partum hemorrhage) OR postpartum haemorrhage) OR post partum haemorrhage)) AND misoprostol)) AND ((((community distribution) OR community)) OR community based))) AND (((((adverse effects) OR adverse outcomes)) OR ((misuse) OR (“Drug Misuse“[Mesh] OR “Prescription Drug Misuse“[Mesh]))) OR ((((skilled delivery) OR institutional delivery) OR “Delivery, Obstetric“[Mesh]) OR delivery))”.

Moreover, a combination of terms, including ‘misoprostol’; ‘misuse’; ‘adverse outcomes’;’ fear of diversion of facility birth’; ‘misconceptions’; ‘misperceptions’; ‘post-partum hemorrhage’ (and variations i.e. ‘post-partum hemorrhage’, ‘postpartum hemorrhage’); ‘community-based maternal’; ‘maternal health interventions’; ‘maternal mortality’; and ‘low-income setting’, ‘developing country’, ‘resource-poor setting’ have been used to identify the required literature from Popline and Google Scholar.

First, any research output with the above-mentioned terms in either the title or abstract of the article was downloaded, and then a combination of these terms was also used to download more resources.

### Critical appraisal

The methodological quality of each study was assessed using the Joanna Briggs Institute (JBI) critical appraisal checklists for different study designs as appropriate [28–30]—to assess the methodological quality of studies and to determine the extent to which included studies have addressed the possibility of bias in its design, conduct, and analysis. Two review authors (GT and MY) independently did appraising the quality of each study included and discrepancies between scores were resolved through discussions.

The quality of the studies was assessed based on the core items recommended for the assessment of methodological quality. To obtain an overall quality score, publications scored “1” point for each item fully met and “0” for none or very little information reported. Items were given equal weights and a percentage score was generated. Studies that scored 75% or more were categorized as high quality, scores in the range of 50–74% were ranked as medium, and scores less than 50% were rated as poor.

Moreover, standard review protocol, Preferred Reporting Items for Systematic and Meta-Analysis for Scoping Reviews (PRISMA-ScR) checklist, was followed to establish minimum information that should be included when reviewing and reporting [31]. The protocol, however, was not registered in any databases.

### Data extraction and analysis

**Fig. 1:**
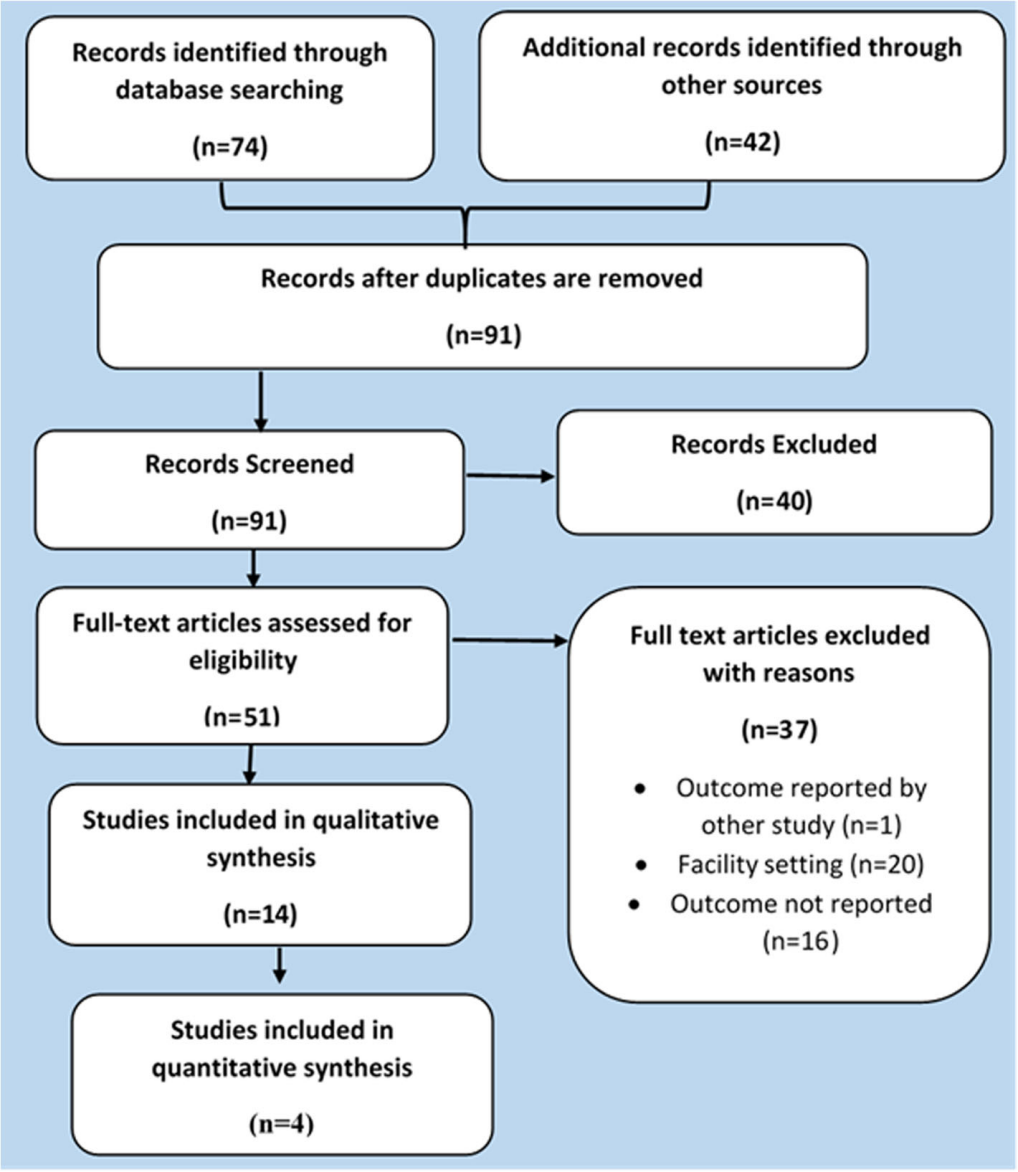
Study flow diagram

The form for abstracting data from reviewed literature was designed and review team members agreed on the contents of the form. Two reviewers (GT and YT) read each identified literature and populated the sheet designed for the purpose. The Preferred Reporting Items for Systematic Reviews and Meta-Analyses (PRISMA) diagram (Fig. 1) was used for the selection of articles to be used in this scoping review.

Facility delivery rate, misuse, adverse effects from misuse of the drug, and misconceptions on the use of misoprostol and fear of diversion of facility delivery to home delivery because of misoprostol’s access to mothers were the main points considered in this scoping systematic review.

A narrative synthesis was used to analyze and interpret the findings in which quantitative and qualitative syntheses are integrated. Descriptive information about the eligible studies was summarized using text and tables. Findings from the quantitative resources were narrated thematically followed by findings of qualitative resources. For intervention studies, a random-effects meta-analysis model [32, 33] was used to pool the estimates of prevalence of facility birth, accounting for the variability among studies using Stata v15 [34]. The results were presented as average treatment effects (odds ratio) with 95% confidence intervals.

## Results

### Description of studies

Table 1 presents the characteristics of the studies included in this review. Fourteen studies were included in the review. Seven of the studies were from Africa and the remaining seven were from Asia. Three qualitative studies [12, 26, 42], seven observational studies [35–37, 40, 43–45], and four experimental or quasi-experimental studies [38, 39, 41, 46] were included in this review. All studies were published from 2006 to 2018.

**Table 1:** Characteristics of included studies

Study ID	Country	Study design	Objectives	Description of the intervention	Outcome
Geller 2014 [35]	Ghana	Facility-based study: before and after intervention comparison	To assess whether community distribution of misoprostol was safe and acceptable To assess whether community distribution of misoprostol was feasible to prevent postpartum hemorrhage if deliveries occur outside health facilities	- Midwives provided misoprostol to expecting women who came for antenatal care during third trimester - Midwives, nurses, community health workers (CHWs), and the study team were trained to deliver educational messages to women, with pictorial flip charts explaining drug safety and administration; - Community sensitization activities regarding safe motherhood, the importance of skilled delivery, and safe storage and use of the drug; - Home visits to pregnant women by CHWs and other members of the study team further emphasized educational messages	- Misuse of misoprostol was not reported in the study - Misoprostol distribution was not found to encourage home deliveries in rural Ghana - Household surveys showed that deliveries with skilled providers increased from 30 to 69%.
Haver 2016 [36]	Afghanistan	Pre- and post-intervention household surveys in 20 districts	To assess whether or not third-trimester distribution of misoprostol would result in adverse events related to child delivery	- CHWs educated and counseled community to raise their awareness on misoprostol - CHWs involved influential people and councils in awareness-raising activities - Misoprostol was distributed to pregnant women in advance during antenatal care - The study/ project provided support to health facilities to carry out clean and safe delivery	- Uterotonic coverage in the community increased by 24% points after intervention - Only 1 woman (out of 7399) reported taking misoprostol before prescribed time (the birth of her newborn) - Misoprostol distribution did not result in any maternal death - The proportion of women who gave birth in a facility increased from 50.2 to 60.8% before and after intervention respectively
Rajbhandari 2010 [37]	Nepal	Before- and after-intervention household survey	- To assess whether advance distribution of misoprostol at community reduces (prevents) PPH occurrence and maternal mortality	- Identified and trained community volunteers to distribute misoprostol at community level - Volunteers educated and counseled pregnant women on prenatal care - Volunteers made home visits to pregnant women and distributed misoprostol at term - Volunteers made postnatal home visits and checked use of misoprostol and any adverse outcomes.	- The proportion of women who had a vaginal delivery who took misoprostol after delivery rose from 11.6% before intervention to 74.2% after intervention - The proportion of women who delivered in a health facility increased from 10.9% at baseline to 14.8% at end line
Sanghvi 2010 [38]	Afghanistan	Community-based: Non-randomized control trial	- To assess whether community distribution of misoprostol was safe and acceptable - To assess whether community distribution of misoprostol was effective and feasible to prevent postpartum hemorrhage	- CHWs made 3 home visits to pregnant women in control and intervention locations - CHWs educated women and support groups in the family on birth preparedness, PPH, facility delivery and postnatal care In addition, in intervention areas - CHWs oriented women and support groups in the family on misoprostol use for PPH prevention and its correct use - CHWs provided misoprostol and visual aids to women after orientation - CHWs made postnatal visits at the woman’s house and check use of misoprostol	- All women in the intervention areas took misoprostol correctly - Uterotonic coverage in the intervention areas increased to 92% while it was 25% in control group - Adverse outcomes were lower in intervention group - 92% of women in the intervention group said they would use misoprostol in the future - A statistically significant difference in the proportion of women delivering in a health facility reported i.e. 21% among intervention group and 18% among control group (*p* < 0.001).
Weeks 2015 [39]	Uganda, Mbale District	Community-based study: placebo-controlled randomized trial	To assess whether self-administration of misoprostol by pregnant women at home was safe and effective	- Women were randomly allocated to either intervention or control group - Women’s hemoglobin level was measured before intervention was provided - Intervention group received misoprostol 600mcg while control groups were provided placebo - Women were counseled on how to use the tablets and provided with the tablets to take at home after birth if birth happens at home - Women’s hemoglobin level was measured on 5th day after delivery	- Only 2 women self-administered the intervention before delivery despite they were told not to do so - More women had experienced shivering and fever among women who took misoprostol compared to placebo group (*p* > 0.05) - Facility delivery: 56.5% in the misoprostol group vs 58.2% in the placebo group (*p* > 0.05)
Smith 2014 [40]	Liberia	Longitudinal observational study	To evaluate whether antenatal distribution of misoprostol was feasible, safe and effective for PPH prevention	- Trained traditional midwives were trained as CHWs to educate and women on misoprostol use - CHWS distributed misoprostol at home - Misoprostol use was assessed at home after delivery	- Only 3(1.1%) women took misoprostol before delivery - Routine data showed that facility delivery increased from 82 during the comparison period (same period in the previous year) to 108 during the intervention period
Ononge 2015 [41]	Uganda	Cluster RCT	- To assess whether misoprostol distribution to pregnant women to administer at home (if she decided to deliver at home) during antenatal care reduces PPH	- Women were offered misoprostol at 28+ weeks of gestation during antenatal care - They were counseled on how to take misoprostol if they delivered at home	- Taking misoprostol before delivery was not reported. - Misoprostol use did not affect postpartum anemia, uterotonic use - Misoprostol use did not affect facility births (85.4% Intervention group vs 87.5% in Control group)
Durham 2018 [42]	Lao People’s Democratic Republic	Qualitative study	- To explore contextual factors that were linked to acceptability misoprostol and whether there was a need to distribute misoprostol at community level for prevention of PPH	- No intervention was done - Interviews were conducted with stakeholders at different levels	All informants stressed on the need for recognized that community distribution of misoprostol as a solution to reduce PPH
Spangler et al. 2014 [26]	Ethiopia	Qualitative study	- To assess decision-makers’ understanding of Ethiopia’s health policy with regard to community-based use of misoprostol for PPH prevention	NA	- Decision-makers had different views and lacked clarity on national policy for community-based distribution of misoprostol for PPH prevention.
Wells et al. 2016 [12]	Ethiopia, Ghana,	Desk review and qualitative methods	- To assess what models existed and implemented to ensure access to misoprostol at community level in Ethiopia, Ghana, and Nigeria	NA	- Care providers’ and decision-makers’ lacked trust in women’s ability to use misoprostol correctly - Care providers’ and decision-makers’ believed that women might use misoprostol pills for abortion - Care providers and decision-makers feared that women might “misuse” misoprostol before delivery - Care providers and decision-makers feared that providers inappropriately might use misoprostol for labor induction and/or abortion
Sibley 2014 [43]	Ethiopia	Household survey and record reviews	- To assess misoprostol use over a period of time - To assess women’s awareness and use of misoprostol and factors associated with its use (before and after a project)	- Trained community health development agents to hold meetings with pregnant women and their caregivers at home. - Community health development agents educated women and distributed misoprostol tablets in the intervention areas i.e. through HEWs (in Amhara) and TBAs (in Oromia)	- Receipt of misoprostol during pregnancy did not affect place of delivery (OR = 0.64; 95% CI, 0.35–1.19, *p* > 0.05). - Very few women took misoprostol before delivery (~ 2%)
Rajbhandari 2017 [44]	Nepal	Mixed methods program evaluation	- To assess whether distribution of misoprostol during antenatal care during a project was effective or not	- No intervention - Household interviews with women who had given birth in the last 12 months in different geographic locations	- Increased awareness of misoprostol use for PPH prevention among women - 96% of community health volunteers said they provided misoprostol to prevent PPH - Misoprostol use did not decrease institutional delivery; - No report that misoprostol was used for any other purpose (including labor induction and abortion). - The majority of those who did not use their advance misoprostol returned it after the birth and most others either threw it away or kept it.
Parashar 2018 [45]	India	Cross-sectional program evaluation	- To develop a framework to assist with designing and implementing community-based distribution of misoprostol	- Pregnant women were more likely to deliver at home (based on criteria) were provided with misoprostol in the 8th month of pregnancy - Pregnant women were counseled about how to use misoprostol if they delivered at home	- Facility delivery increased from 11 to 57% within six months of implementation
Derman 2006 [46]	India	RCT	- To assess whether oral misoprostol could be an alternative drug to oxytocin for PPH prevention	- Auxiliary nurse midwives were trained for 5 days on implementation protocol; attended deliveries; and followed mothers and their newborns postpartum for 6 weeks. - Midwives attended deliveries and administered misoprostol in intervention group and placebo in control group and measured blood loss	- PPH significantly decreased among women who took misoprostol compared to placebo (*p* < 0.001) - Women who took misoprostol were less likely to need referral for emergency care at another hospital (*p* < 0.05) - Women who took misoprostol has higher chance of having transient shivering (*p* < 0.05) - The chance of having nausea, vomiting or diarrhea did not increase due to misoprostol (*p* > 0.05)

Interventional activities in observational and experimental studies included training to health workers, antepartum and/or postpartum home visits, identification of pregnant women, provision of prenatal education, community sensitization, and distribution of 600 μg misoprostol to women.

### Methodological quality of included studies

According to the JBI quality appraisal tool, two of the RCTs scored high quality (88%) and a quasi-experimental study scored medium (61%). On the other hand, the cluster RCT study included scored low (46%) where it had baseline imbalances as well as lacked masking of study of participants, personnel, and assessors [41]. All experimental and quasi-experimental studies provided adequate information about random sequence generation as well as thorough description of the interventions.

Overall, the seven cross-sectional studies scored medium quality (70%) in which most lacked strategies to deal with confounding as well as some lacked appropriate use of statistical methods of analysis. Likewise, all qualitative studies scored medium (65%) in which they are subjected to reporting bias in which philosophical perspectives as well as researchers’ experiences, beliefs, wishes, attitudes, culture, views, and personality not stated which might bias analysis and reporting.

The results of our review are presented under three sections: 1) diversion of facility birth, 2) misuse, for purposes of either abortion or labor induction/augmentation, and 3) adverse events from misuse.

### Diversion of facility birth

**Fig. 2:**
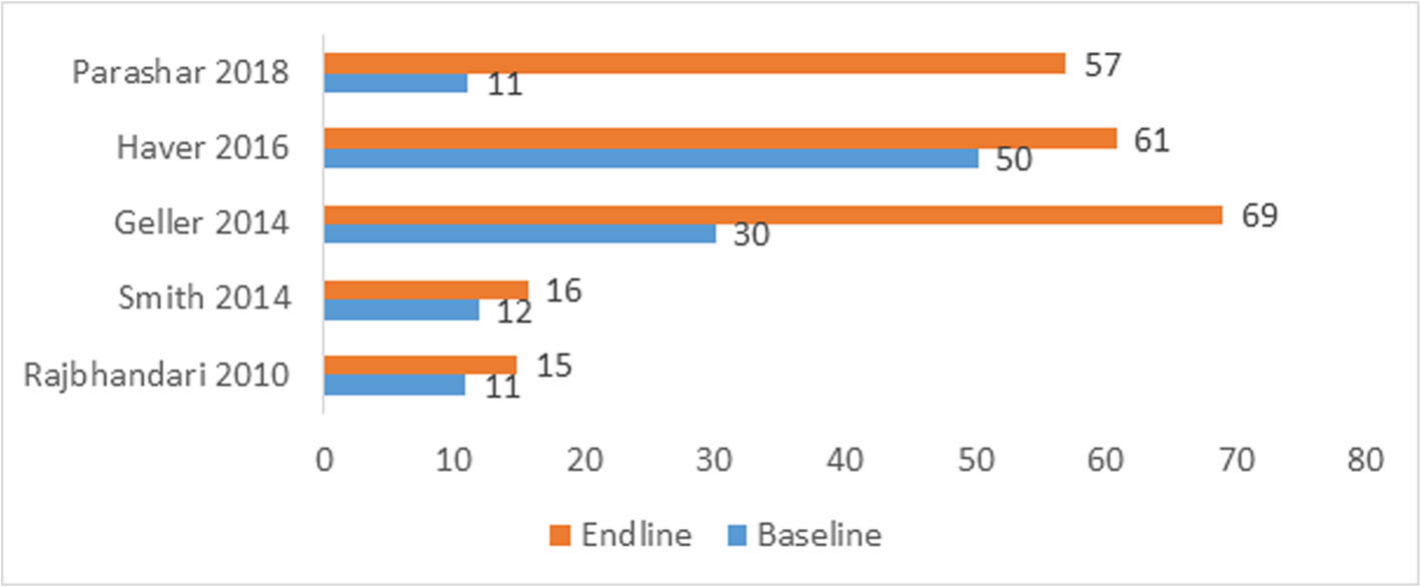
Changes in facility delivery rate before and after the intervention

Eleven studies (five observational before-after studies, four experimental or quasi-experimental trials, and two qualitative study) reported on the impact on facility birth as the outcome [26, 35–42, 45, 46]. All five before-after household surveys reported increased facility delivery coverage after the intervention: four percentage points increase in Nepal [37] and Liberia [40], 11% points in Afghanistan [36], 39% points in Ghana [35], and 46% points in India [45] at the end of the intervention when compared to the baseline (Fig. 2).

A quasi-experimental study in Afghanistan demonstrated an increase of 3.3 percentage points in facility birth rates comparing between the intervention and control areas (*p* < 0.001); while a RCT in India showed a decrease of 1.6 percentage points (*p* > 0.05) and two cluster randomized trials in Uganda showed a decrease of 1.5 and 2.1 percentage points (*p* > 0.05) in facility birth rates, comparing between the intervention and control areas [38, 39, 41]. The pooled analysis involving 7564 women, from four of the studies, revealed that there is no significant difference in facility delivery among the advanced distribution of misoprostol and control groups [OR 1.011; 95% CI: 0.906–1.129] (Table 2).

**Table 2:** Comparison of facility delivery rates between the intervention and control areas

Study	Facility delivery rate (%)		OR	[95% CI]		% Weight
	Intervention	Comparison				
Sanghvi 2010	21.4	18.1	1.229	1.023	1.477	35.93
Weeks 2015	56.5	58.0	0.940	0.697	1.269	13.52
Ononge 2015	85.4	87.5	0.834	0.647	1.075	18.80
Derman 2006	53.2	54.8	0.937	0.770	1.139	31.76
I-V pooled OR			1.011	0.906	1.129	100.0

A qualitative study among health professionals in Laos also indicated that community distribution of misoprostol, for the prevention of PPH, is acceptable to community members and stakeholders and it is a feasible interim solution until access to facility birth is improved. The study recognized misconceptions as barriers that might hinder community-based distribution of misoprostol [42]. Another study in Ethiopia reported regional differences in understanding the implementation strategy of misoprostol and concern among policymakers that distribution of misoprostol will be seen as encouraging home birth [26].

### Misuse

A program evaluation report in Nepal showed that there was no evidence to suggest that misoprostol distributed for the purpose of the prevention of PPH is being misused for labor induction or pregnancy termination [44]. Moreover, as presented in Table 3, in the community-based distribution of misoprostol programs, administration of misoprostol before delivery was reported in less than 2% (*n* = 17) among seven studies involving 11,108 mothers [35, 36, 40, 41, 43].

**Table 3:** Percent of women who took misoprostol before delivery

Study ID	Country	%	n	N
Geller 2014	Ghana	0.00	0	102
Ononge 2015	Uganda	0.00	0	2057
Haver 2016	Afghanistan	0.01	1	7399
Weeks 2015	Uganda	0.29	2	700
Smith 2014	Liberia	1.10	3	265
Sibley 2014	Ethiopia	1.80	11	585
Total			17	11,108

A cluster randomized controlled trial in Uganda [41] and an operations research in Ghana [35] reported that no woman took misoprostol before their babies’ birth. Another before-after study in Afghanistan reported that only 1 out of 7399 women in the study took misoprostol before the birth of her newborn [36]. Similarly, according to a trial in Uganda, only 2 out of 700 women took tablets before delivery [39]. In Liberia, only 3 of 265 women took misoprostol prior to giving birth [40]; while in Ethiopia, less than 2% of women took the tablets before birth [43] (Table 3).

Evidence also shows that most women used the misoprostol pills as instructed [35, 37, 38]; unused doses were returned after birth to the point of distribution; and most others either threw it away or kept it [35, 44]. However, qualitative studies in Ethiopia identified, lack of trust in women’s capabilities to use misoprostol correctly [12] and fear of misuse [12, 26], as a problem limiting the expansion of the program.

### Adverse effects of misuse

Three studies reported minor adverse effects following misoprostol administration [38, 39, 46]. However, no adverse outcomes of misuse were reported in either of the studies reviewed.

## Discussions

This review shows that community-based distribution of misoprostol programs have demonstrated increase of facility delivery coverage after the intervention in observational studies and no significant difference of facility delivery coverage in experimental and quasi-experimental studies among the misoprostol and control groups. The studies reviewed also found very few instances of administration of misoprostol before delivery, and no adverse outcomes because of misuse. While some studies have illustrated a concern held by policymakers and provider about misoprostol misuse, diversion of facility birth, and adverse effects of its misuse [12, 22, 26]; this scoping review showed that, so far, community-based distribution of misoprostol has not negatively impacted facility birth rates (in fact some studies show an increase in facility delivery) and has not resulted in misuse of the medication for uses other than PPH prevention. Accordingly, there is no evidence that substantiates the fear of misoprostol misuse, diversion of facility birth, and other adverse effects of its misuse. As is evident from a qualitative study in Ethiopia [26], these misconceptions arise from the health providers’ perceptions rather than the actual behavior of women using community-distributed misoprostol.

In addition, evidence shows that misoprostol is safe and effective for preventing and treating PPH in remote settings where both oxytocin and timely transfer to higher-level care are not available [21, 25, 47]. Previous studies also report that community health workers or other lower-level workers are able to safely administer misoprostol [16, 42]. Women were found to have no major problem of misusing the drug and it was found to be acceptable by them [16]. Another rapid review of the literature showed that distribution of misoprostol in advance of delivery by lay health workers for self-administration was feasible and acceptable at all levels—end-user, health system, community, and policy [20, 25].

Concerns by policymakers about misoprostol distribution at community level, often unsupported by available evidence [25], impedes the strategy being translated into effective policies, programs, and practice. Concerns primarily include fear of women using misoprostol for inducing abortion or labor, and diversion of facility birth to home deliveries [25, 39, 48]. In addition to policymaker resistance, there is a range of other barriers that impede access to a uterotonic for prevention of PPH for every woman. Barriers include service delivery challenges, supply and procurement, financial, national and global policy environments, and factors more closely connected to the end-user [49]. These implementation barriers represent important threats to any community-based misoprostol distribution program, and most of these barriers are common health system weaknesses in many LMICs [20].

Community-based distribution of misoprostol is a compelling strategy to be implemented parallel to strengthening healthcare facilities to increase safe institutional deliveries [22, 25] and ensuring universal access to uterotonics for every woman. A review by Hobday et al. recommends simultaneously promoting facility delivery and strengthening health systems to avail misoprostol at the community level [16]. Community distribution of misoprostol is thus a complementary strategy for increasing the availability of misoprostol and actively promoting facility births through increasing contact with pregnant women. Increasing interaction with pregnant women also offers the opportunity to promote early care-seeking and referral during pregnancy [20]. As such, community-based distribution of misoprostol programs should include the promotion of facility-based birth [4, 35, 42] as a critical intervention. Successful implementation of misoprostol distribution can be facilitated by creating an enabling environment through supportive policies, designing a formal plan for supplies, task shifting strategies, and appropriate use of guidelines and protocols [27]. Moreover, strong leadership and political commitment, training, and community mobilization were identified as critical success factors [20].

This study provides critical documentation of evidence to support policymakers and program managers to develop national policies and strategies for the implementation of community-based distribution of misoprostol to prevent PPH and reduce maternal mortality. It also highlights that rates of administration of misoprostol before delivery and adverse outcomes of such misuse are very low, especially when compared to the grave risks women can encounter without access to uterotonics. As such, community-based distribution of misoprostol is an appropriate strategy to be implemented while working towards achieving facility delivery as the norm.

National guidance and evidence-based policies on misoprostol distribution initiated by higher levels of the health system can facilitate reassuring reluctant policymakers and providers who hold persistent, but unfounded, fears of misuse and negative consequences. Creating opportunities for reflective discussions or policy dialogue is thus important for virtuous public health practice.

This review has some limitations. First, there may be possibility of missing some relevant studies due to the inclusion of only published studies and exclusion of studies published in a language other than English. Second, we found a small number of articles meeting the inclusion criteria and few rigorous studies directly investigated the negative effect of community availability of misoprostol on institutional delivery, misuse and adverse effects from misuse as a primary outcome. Accordingly, we could not be able to combine all the results in a meta-analysis and show pooled estimates.

## Conclusions

Community-based distribution of misoprostol programs have been associated with an increase in coverage of facility-based births. This review found very few instances of administration of misoprostol before delivery, and no adverse outcomes of misuse in any of the studies reviewed.

Fears of misuse of misoprostol and increased adverse pregnancy outcomes if distributed at community level are not supported by evidence. Therefore, community-based distribution of misoprostol can be an appropriate strategy for reducing maternal deaths caused by postpartum hemorrhages, especially in resource-limited settings where many deliveries take place outside of health facilities.

## Data Availability

The datasets used and/or analyzed during the current study are available from the corresponding author on reasonable request.

## Abbreviations

AMTSL: Active Management of the Third Stage of Labor
CHW: Community Health Workers
JBI: Joanna Briggs Institute
MoH: Ministry of Health
PPH: Postpartum Hemorrhage
PRISMA: Preferred Reporting Items for Systematic Reviews and Meta-Analyses
RAC: Research Advisory Council
RCT: Randomized Controlled Trial
RMNCAH-N: Reproductive, Maternal, Newborn, Child, Adolescent Health, and Nutrition
WHO: World Health Organization
